# Rapid functional genetics of the oligodendrocyte lineage using pluripotent stem cells

**DOI:** 10.1038/s41467-018-06102-7

**Published:** 2018-09-13

**Authors:** Angela M. Lager, Olivia G. Corradin, Jared M. Cregg, Matthew S. Elitt, H. Elizabeth Shick, Benjamin L. L. Clayton, Kevin C. Allan, Hannah E. Olsen, Mayur Madhavan, Paul J. Tesar

**Affiliations:** 1 Department of Genetics and Genome Sciences, Case Western Reserve University School of Medicine, 10900 Euclid Avenue, Cleveland, OH 44106 USA; 2 Department of Neurosciences, Case Western Reserve University School of Medicine, 10900 Euclid Avenue, Cleveland, OH 44106 USA

## Abstract

Oligodendrocyte dysfunction underlies many neurological disorders, but rapid assessment of mutation-specific effects in these cells has been impractical. To enable functional genetics in oligodendrocytes, here we report a highly efficient method for generating oligodendrocytes and their progenitors from mouse embryonic and induced pluripotent stem cells, independent of mouse strain or mutational status. We demonstrate that this approach, when combined with genome engineering, provides a powerful platform for the expeditious study of genotype–phenotype relationships in oligodendrocytes.

## Abstract (web summary)

The isolation and propagation of oligodendroglial cells from postnatal animals can be impractical for functional genetic studies. This study highlights the potential of a new approach to rapidly generate oligodendrocytes and their progenitors from mouse embryonic and induced pluripotent stem cells, independent of mouse strain or mutational status.

## Introduction

Oligodendrocytes generate myelin, a multilaminar structure that allows saltatory propagation of action potentials in the central nervous system (CNS). The functional importance of oligodendrocytes is underscored by numerous neurological diseases characterized by myelin loss or dysfunction, including multiple sclerosis (MS), neuromyelitis optica, leukodystrophies (e.g., Pelizaeus–Merzbacher disease), and mental health disorders such as schizophrenia^1,2^. A myriad of genes, regulatory elements, and single-nucleotide polymorphisms have been associated with oligodendrocyte dysfunction, but reverse genetics has not kept pace due to the cost and time of generating mutant mice and the lack of technology required to genetically manipulate primary oligodendrocytes^3^.

To facilitate more rapid studies into the molecular mechanisms that underlie oligodendrocyte function and myelin disease, we report a new method for generating pure and highly scalable populations of myelinogenic oligodendrocytes and their progenitor cells (oligodendrocyte progenitor cells (OPCs)) from pluripotent cell sources including mouse embryonic stem cells (mESCs) and induced pluripotent stem cells (iPSCs). Our previous method of generating OPCs from pluripotent mouse epiblast stem cells relied on a starting cell type that is challenging to grow, less accessible, and difficult to genetically manipulate^4^. In contrast, our new method is highly efficient and universally reproducible across pluripotent stem cell lines from any wild-type or mutant genetic background, as well as from lines purposefully edited with CRISPR-Cas9 nuclease or other methods of genome engineering. Given the widespread accessibility of mESCs and iPSCs, this new protocol can be combined with existent methodologies such as CRISPR-Cas9 technology to enable in vitro molecular and cellular phenotyping of OPCs and oligodendrocytes with defined genotypes in <3 weeks.

## Results

### Generation of myelinogenic OPCs from patterned mESCs

**Fig. 1:**
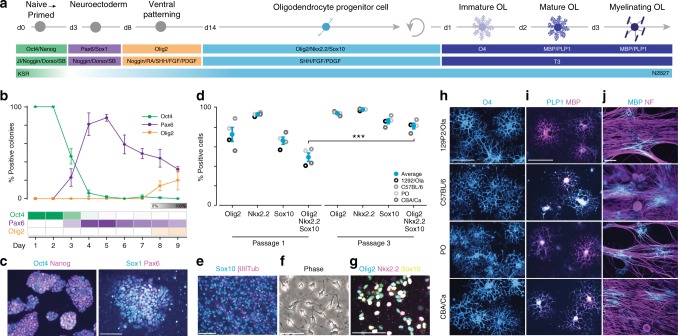
Reproducible generation of OPCs and oligodendrocytes from mESCs. **a** Graphical overview of the differentiation time course for generating OPCs and stage-specific oligodendrocytes from mESCs. **b** Quantification of immunocytochemistry for stage-specific markers demarcating the transition from pluripotency (Oct4) to neuroectoderm (Pax6) to ventral neural tube (Olig2) over 9 days. *n* = 4 independent biological replicates (mESC lines) with >25 colonies scored per cell line. Data are represented as means ± SEM. **c** Representative immunofluorescent images of starting mESCs (Oct4 and Nanog) and day 5 neuroectoderm (Pax6 and Sox1). Scale bar, 50 µm. **d** Quantification of OPC-defining transcription factors Olig2, Nkx2.2, and Sox10 at passages 1 and 3 of the differentiation protocol. *n* = 4 independent mESC lines; >114,500 cells scored per cell line. Data are represented as means ± SEM. ***P value <0.001; unpaired *t* test. **e** Immunofluorescent image of passage 1 cultures stained for Sox10, an OPC marker, and βIII-Tubulin, a marker of neurons. Scale bar, 50 µm. **f** Representative phase contrast image of mESC-derived OPCs exhibiting a canonical bipolar morphology. Scale bar, 50 µm. **g** Immunostained image of passage 3 mESC-derived OPCs co-expressing Olig2, Nkx2.2, and Sox10, canonical OPC transcription factors. Scale bar, 50 µm. **h** Cell surface immunostaining of the immature oligodendrocyte marker O4, after treatment with T3. Scale bar, 50 µm. **i** Representative images of differentiated OPCs immunostained for mature oligodendrocyte markers MBP and PLP1, 72 h post treatment with T3. Scale bar, 50 µm. **j** Representative images of OPC/DRG co-cultures stained for MBP and neurofilament (NF) at day 10. Scale bar, 50 µm. Unless otherwise noted, images presented in Fig. 1 are derived from the CBA/Ca mESC line, and are representative of results obtained with C57BL/6, PO, and 129P2/Ola lines, which are shown separately in Supplementary Fig. 2

We initially selected four previously isolated germline-competent male mESC lines derived from independent mouse strains 129P2/Ola, C57BL/6, PO, and CBA/Ca^5^. Prior to beginning the current studies, ES cell cultures were karyotyped (see Methods) and confirmed to express canonical markers of pluripotency, Oct4 and Nanog (Supplementary Figs. 1a, b and 2a). All four mESC lines were then differentiated to OPCs and oligodendrocytes using stage-specific small molecules and growth factors that mimic signaling events known to specify oligodendrocyte fate during development (see Fig. 1a for overview and Methods for the detailed protocol)^4,6–14^.

First, naive mESCs were transitioned to primed epiblast-like cells by culturing in suspension as spheres in the presence of a small-molecule inhibitor of the Janus kinase (JAK)/signal transducers and activators of transcription pathway^6^. Cells were then specified to the neuroectodermal lineage using small molecules and recombinant proteins to inhibit both Activin/Nodal and bone morphogenetic protein signaling pathways^15^. By day 5 of differentiation, cells robustly downregulated the pluripotency marker Oct4 and upregulated the early neuroectodermal lineage marker Pax6 (Fig. 1b, c and Supplementary Fig. 2b). Subsequent treatment with retinoic acid and sonic hedgehog (SHH) stimulated downregulation of Pax6 and emergence of Olig2 expression, a domain marker of the ventral developing neural tube from which OPCs are first specified in vivo (Fig. 1b–g)^16^.

To facilitate outgrowth, expansion, and maturation of OPCs, day 9 ventralized spheres were seeded on polyornithine and laminin-coated culture plates (termed passage 0). Neuronal axons rapidly extended from the attached spheres followed by migratory and proliferative early OPCs expressing Olig2 and Sox10 (Fig. 1d, e, g and Supplementary Fig. 2c). Passaging of these cultures in the presence of fibroblast growth factor (FGF), platelet-derived growth factor (PDGF), and SHH enabled selective enrichment and continued maturation of OPCs co-expressing Olig2, Sox10, and Nkx2.2. At passage 1, 49 ± 4.32% of cells co-expressed all three of these OPC-defining transcription factors and adopted a bipolar morphology typical of bona fide OPCs (Fig. 1d–f, g and Supplementary Fig. 2d) and nearly all cells expressed at least one of the three markers (Fig. 1d). GFAP+ or βIII-Tubulin+ cells, demarcating astrocytes and neurons, respectively, accounted for <2% of the total number of cells at passage 1 (GFAP+, 0.74 ± 0.30%; βIII-Tubulin+, 0.54 ± 0.38%; Supplementary Fig. 2e, f), suggesting that nearly all cells were early-stage OPCs. Within two additional passages, cultures matured to a near-homogeneous population in which 81 ± 2.92% of cells co-expressed Nkx2.2, Sox10, and Olig2 and 93 ± 0.72% of cells expressed two of the three defining OPC markers across all four independent mESC lines without sorting or selection (Fig. 1d). These mESC-derived OPCs could be expanded for an additional 5–7 passages enabling rapid generation of billions of pure OPCs.

To confirm that mESC-derived OPCs could differentiate into terminally mature oligodendrocytes, the cells were treated with thyroid hormone (T3), a known stimulator of oligodendrocyte differentiation, in the absence of PDGF and FGF (Fig. 1a)^14,17^. Within 24–48 h, OPCs exhibited a multi-processed branched morphology and expressed the early oligodendrocyte cell surface antigen O4 (Fig. 1h and Supplementary Fig. 2g). By 72 h, cells took on a highly ramified morphology characteristic of mature oligodendrocytes (Fig. 1i) and expressed mature myelin proteins myelin basic protein (MBP) and proteolipid protein 1 (PLP1) (Fig. 1i and Supplementary Fig. 2g). To assess the capacity of mESC-derived OPCs to associate with and wrap axons (in vitro “myelination”), cells were co-cultured with dissociated dorsal root ganglion (DRG) sensory neurons for 10 days^18^. OPCs gave rise to MBP+ oligodendrocytes with segmented tracts of MBP in close apposition with neurofilament-positive (NF+) DRG axons, indicative of axonal ensheathment and myelinating capability (Fig. 1j). Additionally, enhanced green fluorescent protein (eGFP)-labeled mESC-derived OPCs were injected into the developing brains of 2-day-old athymic nude mice. At 6 weeks post injection, cells exhibited eGFP+ segmented tracts co-localized to NF+ axons (Supplementary Fig. 3a, b). Taken together, these findings show that mESC-derived OPCs give rise to myelinogenic oligodendrocytes in vitro and in vivo.

### Cellular profiling of mutant alleles

**Fig. 2:**
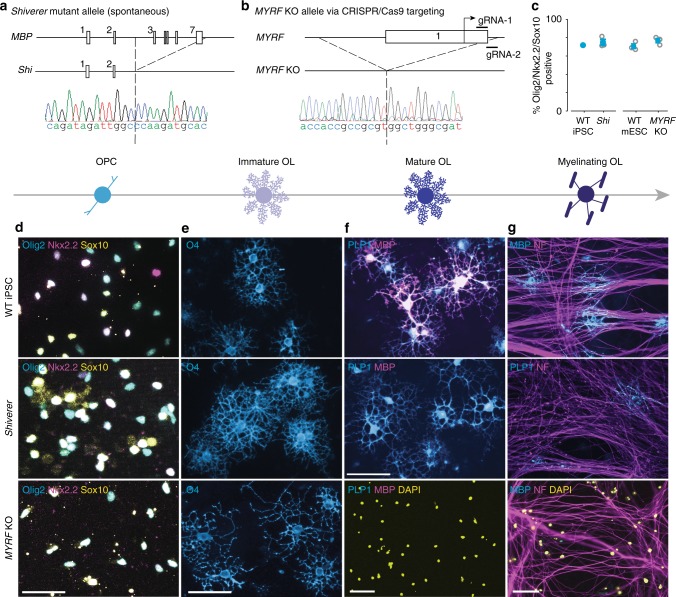
Cellular profiling of spontaneous and purposely generated mutant oligodendrocyte alleles. **a** Diagram indicating that *shiverer* mice harbor a ~20-kilobase (kb) homozygous deletion encompassing exons 2–7 of the MBP gene. A Sanger sequencing trace shows the breakpoint of the *shiverer* deletion. **b** Diagram indicating the location of the two gRNAs designed to target *MYRF*. A Sanger sequencing trace shows the location of the homozygous deletion of exon 1. **c** Quantification of transcription factors Olig2, Nkx2.2, and Sox10 at passage 3 of the differentiation protocol. *n* = 3 *shiverer* cell lines; *n* = 3 replicate wells per cell line; >179,500 cells scored per well. *n* = 3 *MYRF* KO and wild-type (WT) mESC replicate wells per cell line; >700 cells scored per well. Data are represented as means ± SEM. **d** Fluorescent images of WT iPSC, *shiverer*, and *MYRF* KO OPCs expressing canonical OPC markers Olig2, Nkx2.2, and Sox10. Scale bar, 50 µm. **e** Cell surface immunostaining of the immature oligodendrocyte marker O4, after treatment with T3, of WT iPSC, *shiverer*, and *MYRF* KO OPCs. Scale bar, 50 µm. **f** Representative images of differentiated OPCs immunostained for mature oligodendrocyte markers MBP and PLP1, 72 h post treatment with T3 of WT iPSC, *shiverer*, and *MYRF* KO OPCs. Scale bar, 50 µm. **g** Representative images of OPC/DRG co-cultures stained for MBP and neurofilament (NF) at day 10 from WT iPSC, *shiverer*, and *MYRF* KO OPCs stained for PLP1 or MBP after being co-cultured for 10 days with NF+ embryonic rat DRGs. Scale bar, 50 µm

The ability to reproducibly generate OPCs and oligodendrocytes from any mouse wild-type or mutant genetic background allows for the interrogation of cellular and molecular defects underlying spontaneous or engineered mutations within those cells. To validate the power of this approach, we generated iPSC lines from *shiverer* mice, which harbor a large homozygous deletion in the *MBP* gene that results in CNS hypomyelination (Fig. 2a)^19^. Additionally, we used CRISPR-Cas9 in wild-type mESCs (strain C57BL/6) to target myelin regulatory factor (*MYRF*), a transcription factor required for oligodendrocyte differentiation^20^, to generate *MYRF* knockout (KO) mESCs (Fig. 2b). We then differentiated *shiverer* and *MYRF* KO pluripotent stem cell lines to OPCs and observed no difference in differentiation efficiencies compared to the wild-type mESC and iPSC control lines (Fig. 2c, d).

To interrogate more mature cellular phenotypes, wild-type, *shiverer*, and *MYRF* KO OPCs were induced to differentiate into oligodendrocytes either in isolation or in co-culture with DRG neurons. Predictably, wild-type mESC-derived and iPSC-derived OPCs differentiated over 72 h through an O4+ immature oligodendrocyte state (Fig. 2e) into mature PLP1+/MBP+ oligodendrocytes (Fig. 2f), which ensheathed DRG axons (Fig. 2g), indicative of myelination competency. In contrast, *shiverer* and *MYRF* KO cells exhibited stage-specific in vitro cellular phenotypes consistent with their previously defined in vivo pathology^20–22^. *Shiverer* OPCs readily gave rise to morphologically mature PLP1+ oligodendrocytes that failed to express MBP and thus had limited capacity to ensheath DRG axons (Fig. 2f, g). *MYRF* KO OPCs arrested at the immature O4+ stage with no cells expressing mature oligodendrocyte markers or ensheathing DRG axons (Fig. 2e–g). Collectively, these data validate that our method can easily be used to rapidly phenotype oligodendrocyte cellular dysfunction across various genetic backgrounds.

### Molecular profiling of mutant alleles

**Fig. 3:**
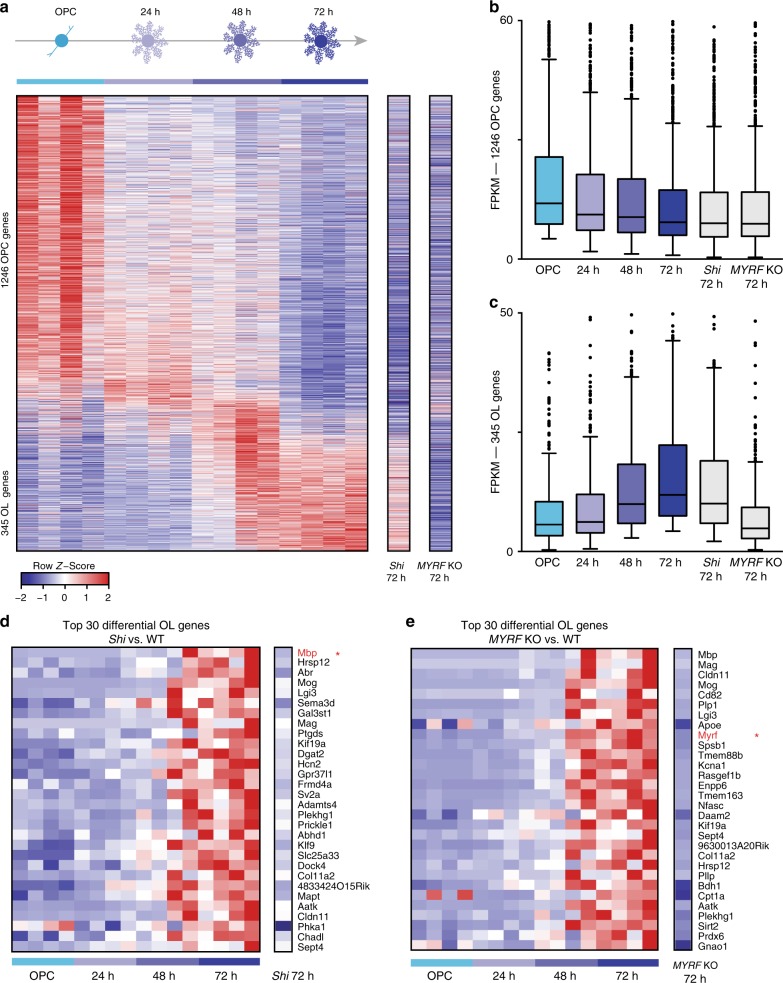
Molecular profiling of spontaneous and purposely generated mutant oligodendrocyte alleles. **a** Row normalized heatmap with genes (rows) sorted by time of maximum (TOM). The four independent WT mESC lines for each time point are shown compared to gene expression of *shiverer* and *MYRF* KO OPCs 72 h post T3 induction. **b** Boxplot of OPC-signature genes prior to T3 induction and after differentiation with T3 for 24, 48, and 72 h. **c** Boxplot of oligodendrocyte-signature genes prior to T3 induction and after differentiation with T3 for 24, 48, and 72 h. **d** Row normalized heatmap of the top 30 dysregulated oligodendrocyte-signature genes for *shiverer* 72 h post T3 induction. Gene expression for the four WT mESC strains for each time point are shown compared to gene expression of *shiverer* OPCs 72 h post T3 induction. Note the lack of MBP expression (red star). **e** Row normalized heatmap of the top 30 dysregulated oligodendrocyte-signature genes for *MYRF* KO OPCS at 72 h post T3 induction. Gene expression for the four WT mESC strains for each time point are shown compared to gene expression of *MYRF* KO OPCs 72 h post T3 induction. Note the lack of MYRF expression (red star)

To establish a temporal scorecard of gene expression from wild-type cells (*n* = 4 independent mESC lines), we performed time of maximum (TOM) analysis of RNA-sequencing (RNA-seq) data to identify genes with stage-specific expression over 72 h of differentiation from OPCs to oligodendrocytes (Fig. 3a and see Supplementary Data 1 for full gene lists). These TOM gene sets showed strong overlap with previous RNA-seq data generated from in vivo isolated cells, suggesting that they define physiologically relevant cellular transitions (Supplementary Fig. 4)^23^. We used stage-specific TOM genes to define molecular correlates of cellular dysfunction observed in *shiverer* and *MYRF* KO cells. RNA-seq profiling of *shiverer* OPCs after 72 h of differentiation showed downregulation of OPC-signature genes and upregulation of oligodendrocyte-signature genes (Fig. 3b–d), with a few exceptions—notably, full-length expression of MBP was not observed, confirming that the *shiverer* mutation precludes proper MBP transcription. In addition, key myelination genes such as *MAG* and *MOG* were downregulated in *shiverer* oligodendrocytes relative to the differentiated control lines, consistent with the late-stage myelination defect identified in our cellular assays (Fig. 3d). RNA-seq profiling of 72 h differentiated *MYRF* KO OPCs showed clear downregulation of the OPC-signature genes and a complete failure to upregulate mature oligodendrocyte-signature genes (Fig. 3b, c–e), with notable absence of *MBP*, *MAG*, *MOG*, *PLP1*, and, as expected, *MYRF* expression (Fig. 3e). This global molecular phenotype indicates that *MYRF* KO OPCs arrest at an early stage of differentiation that is consistent with their failure to progress beyond the immature O4+ oligodendrocyte stage in our cellular assays. Taken together, these results define OPC-specific and oligodendrocyte-specific expression signatures and underscore their utility in in vitro stage-specific molecular profiling of mutant alleles.

## Discussion

In summary, we provide a transformative platform for rapidly generating highly scalable populations of OPCs and oligodendrocytes. Until now, isolation of genotype-specific oligodendrocytes has been confined to pre-existing mouse strains. In contrast, our 3-week differentiation protocol harnesses the amenability of pluripotent stem cells to genetic modification and can be combined with existing technologies such as CRISPR-Cas9 to gain novel insight into developmental biology underlying normal and disease states in the oligodendrocyte lineage. Our method is also amenable to leverage the power of newer functional genetic tools, such as the dTag protein degrader system^24^, to provide previously unprecedented temporal and selective control of any protein during oligodendrocyte development. As novel factors (genes, genetic variants, microRNAs, long noncoding RNAs, and epigenetic signatures) that regulate glial biology and disease continue to be identified, our method can be utilized for first tier proof-of-principle studies in lieu of more expensive and time-consuming approaches (e.g., generation and maintenance of mutant mouse colonies). Moreover, the ability to generate large populations of pure OPCs from normal and disease states provides the scalability necessary for biochemical, epigenetic, and proteomic analyses, and for large-scale genetic and/or chemical screens.

## Methods

### Animal welfare

All animal experiments were performed in accordance with protocols approved by the Case Western Reserve University Institutional Animal Care and Use Committee.

### Isolation of fibroblasts

Mouse embryonic fibroblasts (MEFs) were isolated at embryonic day 13.5 (E13.5) from embryos generated through timed natural matings between *shiverer* homozygous mice (C3Fe.SWV-*Mbp*^*shi*^/J; Jackson Laboratory). Cells were expanded for one passage and cryopreserved for future use. Wild-type tail tip fibroblasts (TTFs) were generated from postnatal day 9 mice (B6CBACa-*A*^*w-J*^; Jackson Laboratory). Tail tips of approximately 2 mm in length were bisected with a sterile scalpel and cultured on a Nunclon-Δ wells under glass coverslips for outgrowth. MEFs and TTFs were isolated and expanded in fibroblast medium, which consisted of DMEM supplemented with 10% fetal bovine serum (FBS; Thermo Fisher), 2 mM glutamax (Thermo Fisher), 1× nonessential amino acids (Thermo Fisher), and 0.1 mM 2-mercaptoethanol (Sigma).

### Production of lentivirus

Lentivirus was generated for pLVX-Tet-On-Puro Advanced, pHAGE2-TetOminiCMV-STEMCCA-W-loxp (kindly gifted from Gustavo Mostoslavsky), and pLV-eGFP plasmids according to the manufacturer’s protocol using the Lenti-X HT Packaging Mix and Lenti-Phos or Cal-Phos Mammalian Transfection Kit or Lenti-X Packaging Single Shots (all from Clontech). The 293T cells (5.0 × 10^4^ cells/cm^2^) (Clontech) were cultured on rat tail collagen I-coated plasticware (BD Biosciences) and transfected 16 h later in Dulbecco's modified Eagle's medium (DMEM) supplemented with 5% FBS, 2 mM glutamax, 1× nonessential amino acids, and 0.1 mM 2-mercaptoethanol (for iPSC generation) or neural medium supplemented with 20 ng/ml FGF2 and 20 ng/ml PDGF-AA (for eGFP+ OPCs). Individual supernatants containing virus were harvested and filtered with a 0.45 µm polyvinylidene difluoride (PVDF) membrane (Millipore) 24 and 48 h later.

### Generation of mouse iPSCs

MEFs or TTFs were seeded at 1.3 × 10^4^ cells/cm^2^ on Nunclon-Δ plates, allowed to attach overnight, and infected with 50/50 (vol/vol) of pLVX-Tet-On-Puro Advanced and pHAGE2-TetOminiCMV-STEMCCA-W-loxp lentivirus supplemented with polybrene (8 µg/ml)^25^. Cells were incubated in lentivirus for 3 h followed by replacing the medium with fibroblast medium supplemented with 2 µg/ml doxycycline (Clontech). Cells were cultured in fibroblast medium supplemented with doxycycline for 3 days after which cells were lifted with 0.25% Trypsin-EDTA (Thermo Fisher) and either frozen or seeded at 3–6 × 10^4^ cells/cm^2^ on Nunclon-Δ plates pre-plated with 4.5 × 10^5^ cells/cm^2^ irradiated MEF (iMEF) feeder layer and cultured in doxycycline-containing mESC medium, which consisted of KO DMEM (Thermo Fisher) supplemented with 5% ES cell-qualified FBS (Thermo Fisher), 15% KO replacement serum (KSR; Thermo Fisher), 2 mM glutamax, 1× nonessential amino acids, 0.1 mM 2-mercaptoethanol, and 10^3^  U/ml leukemia inhibitory factor (LIF; Millipore). Media were changed every other day until colonies started to emerge. Colonies were picked individually, dissociated in 0.25% Trypsin-EDTA (Thermo Fisher), and plated in individual Nunclon-Δ 1.9 cm^2^ wells pre-plated with an iMEF feeder layer and cultured in mESC medium. On the day of picking, the colonies were considered passage 0. Media were changed every day. iPSC lines were expanded to passage 6 before being used for differentiation experiments.

### Generation of *MYRF* KO cells

Two single guide RNAs (sgRNAs) were designed for *MYRF* according to Ran et al.^26^ (crispr.mit.edu) and synthesized by Sigma into their pCMV-Cas9-GFP plasmid to contain sgRNA under the U6 promoter and upstream of Cas9-2A-GFP (GCGCTGCAGCGCTTCTTCGA, *MYRF* sgRNA-Cas9_1 and TTCGAAGGTGAGAGACCGCG, *MYRF* sgRNA-Cas9_2). mESCs were seeded at 3.1 × 10^4^ cells/cm^2^ on an iMEF feeder layer in mESC medium supplemented with 10^3^ U/ml LIF. The medium was changed every day for 3 days. On day 3, cells were dissociated with 0.25% Trypsin-EDTA and 5.6 × 10^6^ mESCs were electroporated with 7 µg of sgRNA-Cas9_1, 7 µg of sgRNA-Cas9_2, and 7 µg of EF1α-cre-IRES-puro using the Amaxa Nucleofector II, nucleofector program A-023. Cells were seeded at 2 × 10^5^ cells/cm^2^ on drug-resistant iMEF (GlobalStem)-coated Nunclon-Δ plates in mESC medium supplemented with 10^3^ U/ml LIF. Twenty-four hours after electroporation, the medium was changed and cultures were fed with mESC medium supplemented with 2 µg/ml puromycin (Thermo Fisher) for 2 days followed by daily medium changes with mESC medium for 6 days. Cells were dissociated and seeded at 5.2 × 10^2^ cells/cm^2^ on iMEF-coated Nunclon-Δ plates in mESC medium supplemented with 10^3^ U/ml LIF. The medium was changed daily with mESC medium for 5 days. Twenty colonies were picked and expanded in mESC medium. PCR was performed on each colony and 12 colonies were selected for Sanger sequencing based on their gel band shift from wild-type control. Sanger sequencing revealed indels in 11 of the 12 sequenced colonies. The colony with the largest homozygous deletion surrounding the start site of exon 1 of *MYRF* (see Fig. 2b) was selected for future studies.

### OPC generation from mouse pluripotent stem cells

All cells were cultured at 37 °C and 5% CO_2_, unless otherwise noted. All cells tested free of mycoplasma. Individual germline-competent, determined by blastocyst injection, male mESC lines (kindly provided by Richard Gardner and Frances Brook, Oxford, UK) were isolated previously from timed natural matings of mice of strains 129P2/Ola (line ID = ESF58/2), C57/BL6 (line ID = ESF75), CBA/Ca (line ID = ESF122), and closed bred PO (line ID = ESF112)^5^. For OPC generation, mESCs and iPSCs were seeded at 3.4 × 10^4^ cells/cm^2^ and maintained on an iMEF feeder layer in mESC medium, supplemented with 10^3^ U/ml LIF 3 days prior to differentiation. On day 0 of the differentiation protocol, mESC or iPSC colonies were passaged free of the iMEF feeder layer by treatment with 1.5 mg/ml collagenase type IV (Thermo Fisher) followed by dissociation to single cells with 0.25% Trypsin-EDTA (Thermo Fisher). iMEF-free mESCs and iPSCs were seeded at 7.8 × 10^4^ cells/cm^2^ on low attachment plates (Sigma) in KSR medium, which consisted of KO DMEM (Thermo Fisher) supplemented with 20% KSR, 2 mM glutamax, 1× nonessential amino acids, 0.1 mM 2-mercaptoethanol. On day 1, the medium was changed and cultures were fed with KSR medium supplemented with 0.2 µM JAK inhibitor I (Calbiochem). On day 2, the medium was changed and cultures were fed with 50/50 (vol/vol) KSR medium and neural medium, which consisted of DMEM/F12 (Thermo Fisher) supplemented with 1× N2 (R&D Systems), 1× B-27 without vitamin A (Thermo Fisher), and 2 mM glutamax and supplemented with 0.2 µM JAK inhibitor. On day 3, the medium was changed and cultures were fed with 50/50 (vol/vol) KSR medium and neural medium supplemented with 0.2 µM JAK inhibitor, 100 ng/ml noggin (R&D Systems), 20 µM SB431542 (Sigma), and 2 µM dorsomorphin (EMD). On days 4 and 5, the medium was changed and cultures were fed with 50/50 (vol/vol) KSR medium and neural medium supplemented with 100 ng/ml noggin, 20 µM SB431542, and 2 µM dorsomorphin. On day 6, the medium was changed and cultures were fed with 50/50 (vol/vol) KSR medium and neural medium supplemented with 100 ng/ml noggin. On days 7 and 8, the medium was changed and cultures were fed with 25/75 (vol/vol) KSR medium and neural medium supplemented with 100 ng/ml noggin, 10 µM all-*trans* retinoic acid (Sigma), and 200 ng/ml SHH (R&D Systems). On day 9, cells were collected and split 1:2 on Nunclon-Δ wells coated with poly(l-ornithine) (Sigma) followed by laminin (Sigma) and cultured in neural medium supplemented with 100 ng/ml noggin, 200 ng/ml SHH, 20 ng/ml FGF2 (R&D Systems), and 20 ng/ml PDGF-AA (R&D Systems). At this point, the cells were considered “passage 0.” On day 10, the medium was changed and cultures were fed with neural medium supplemented with 200 ng/ml SHH, 20 ng/ml FGF2, and 20 ng/ml PDGF-AA. The medium was changed every other day with “day 10” medium. Cells were passaged at 80–90% confluence and seeded at 2.5 × 10^4^ cells/cm^2^. Cultures of mESC-derived or iPSC-derived OPCs were maintained and expanded on Nunclon-Δ wells coated with poly(l-ornithine) followed by laminin in neural medium supplemented with 200 ng/ml SHH, 20 ng/ml FGF2, and 20 ng/ml PDGF-AA. To facilitate enrichment and maturation of the OPC population, mESC-derived or iPSC-derived OPCs were cultured for one passage without FGF2 in neural medium supplemented with 200 ng/ml SHH, 20 ng/ml PDGF-AA, 100 ng/ml noggin, 100 ng/ml insulin-like growth factor 1 (IGF-1) (R&D Systems), 10 µM cyclic AMP (Sigma), and 10 ng/ml NT3 (R&D Systems). OPCs were readily cryopreserved and recovered by freezing in DMEM supplemented with 10% FBS and 10% dimethyl sulfoxide (Sigma).

### Quantification of neural induction

Cell culture suspensions were plated onto Nunclon-Δ wells coated with poly(l-ornithine) and laminin. Plated spheres were cultured in appropriate patterning medium conditions for 8 h followed by fixation with 4% paraformaldehyde (PFA). Cells were stained with antibodies against Oct4, Pax6, and Olig2 (see Immunocytochemistry methods). Twenty-five individual spheres from each of the four wild-type mESC lines were scored for each protein (Oct4, Pax6, and Olig2) on each patterning day (days 1–9). Colonies were scored positive if more than 50% of the cells in the colony expressed the factor.

### OPC differentiation to oligodendrocytes

OPCs were seeded at 2–2.6 × 10^4^ cells/cm^2^ on Nunclon-Δ wells coated with poly(l-ornithine) followed by laminin in supplemented neural medium for 2 days. On day 3, media were aspirated and cells were washed with 1× phosphate-buffered saline (PBS) and cultured in neural medium supplemented with 200 ng/ml SHH, 100 ng/ml noggin, 100 ng/ml IGF-1, 10 µM cyclic AMP, and 10 ng/ml NT3, and 40 ng/ml T3 for 3 days.

### Immunocytochemistry

Cells were prepared for immunostaining by fixation in 4% PFA (Electron Microscopy Sciences) for 15 min and subsequent permeabilization for 10 min with 0.2% Triton X-100 in 1× PBS. Cells were then blocked for non-specific binding with filtered 10% normal donkey serum (Abcam) in 1× PBS for 1 h at room temperature. Primary antibodies were diluted in blocking solution and incubated with the samples overnight at 4 °C. Samples were rinsed with 1× PBS and incubated with the appropriate fluorescently labeled Alexa-Fluor secondary antibodies (Thermo Fisher, 1:500) for 1 h at room temperature. For nuclear staining, samples were incubated with 1 μg/ml 4′,6-diamidine-2′-phenylindole dihydrochloride (DAPI) (Sigma) for 5 min. Primary antibodies used were: Sox10 (R&D Systems, AF2864; 2 µg/ml), Olig2 (Millipore, AB9610 or ProteinTech, 13999-1-AP; 1:1000), Sox1 (R&D Systems, AF3369; 1 µg/ml), Pax6 (Covance, PRB-278P; 0.67 µg/ml), Oct4 (Santa Cruz, SC-5279; 0.4 µg/ml), Nkx2.2 (DSHB, 74.5A5; 4.4 μg/ml), MBP (Covance, SMI-99P; 2 µg/ml), O4 (kindly gifted from Robert Miller; 1:10), PLP1 (kindly gifted from Bruce Trapp; 1:200), Nanog (Abcam, AB21624; 1 µg/ml), and βIII-Tubulin (R&D Systems, MAB1195; 1 µg/ml).

### OPC-DRG co-culture

All cells were cultured at 37 °C and 5% CO_2_, unless otherwise noted. OPC-DRG co-cultures were prepared as described previously^7^. Briefly, DRG neurons were isolated and dissociated from E15 Sprague–Dawley rats and seeded at 7 × 10^4^ cells per 18-mm collagen-coated glass coverslips. DRGs were cultured in medium supplemented with 100 ng/ml of nerve growth factor (Serotec), 2 mM Uridine (Sigma), and 2 mM 5-fluoro-2′-deoxyuridine (Sigma) for 3 weeks. DRGs were washed extensively with 1× PBS before plating 9–10 × 10^4^ OPCs per coverslip. Co-cultures were cultured in neural medium for 10 days before fixation.

### Immunohistochemistry of OPC-DRG co-cultures

Cells were prepared for immunostaining by fixation in 100% ice-cold methanol for 20 min. Cultures were rinsed with 1× PBS and blocked for non-specific binding with filtered 5% normal donkey serum in 0.1% Triton X-100 at room temperature for 1 h. Primary NF antibodies were diluted in 2% normal donkey serum in 0.1% saponin and incubated overnight at 4 °C. Samples were rinsed with 1× PBS and incubated with appropriate fluorescently labeled Alexa-Fluor secondary antibody (Thermo Fisher; 1:500) for 1 h at room temperature. Cultures were rinsed with PBS and blocked with 2% normal donkey serum in 0.1% saponin for 1 h at room temperature. Primary PLP1 and MBP antibodies were diluted in 2% normal donkey serum in 0.1% saponin and incubated overnight at 4°C. Samples were rinsed with 1× PBS and incubated with appropriate fluorescently labeled Alexa-Fluor secondary antibodies (Thermo Fisher; 1:500) for 1 h at room temperature. For nuclear staining, samples were incubated with 1 μg/ml DAPI (Sigma) for 5 min. Primary antibodies used were: MBP (Abcam, ab7349; 1:100), PLP1 (kindly gifted from Bruce Trapp; 1:100), and NF cocktail (Covance, SMI-311 and SMI-312; 1:100).

### Transplantation of OPCs

OPCs were seeded at 2.9 × 10^4^ cells/cm^2^ on Nunclon-Δ wells coated with poly(l-ornithine) followed by laminin, allowed to attach overnight, and infected with 100 (vol) of pLV-eGFP lentivirus supplemented with protamine sulfate (8 µg/ml; Sigma). Cells were incubated in lentivirus for 4 h followed by the addition of neural medium. After an additional 20 h of incubation, the medium was replaced with neural medium supplemented with 20 ng/ml FGF2 and 20 ng/ml PDGF-AA. OPCs were passaged before transplantation. On postnatal day 2, NU/J (Jackson Laboratory) mice were cryoanesthetized and injected with 5 × 10^4^ eGFP+ OPCs in 0.5 μl Hank's balanced salt solution (Thermo Fisher) at a depth of 1.5 mm, 1 mm lateral of the sagittal suture and midway between bregma and lambda^27^. On postnatal day 42 (6 weeks), transplanted mice were anesthetized with isoflurane to effect and transcardially perfused with 4% PFA (Electron Microscopy Sciences) followed by incubation in 4% PFA for 16 h at 4 °C. Tissue was then washed in 1× PBS followed by incubation in 30% sucrose (Sigma) at 4 °C. Brains were embedded in optimum cutting temperature formulation (Tissue-Tek), sectioned at 20 μm on a Leica CM 1950 crytostat, and mounted on SuperFrost Plus slides (Fisher Scientific). For fluorescent staining, slides were incubated in permeabilization buffer (0.25% Triton X-100, 2% donkey serum, and 1× PBS) and NF cocktail (Covance, SMI-311R, SMI-312; 1:500) and GFP (Thermo Fisher, A11122; 1:500) primary antibodies overnight. Slides were washed with 1× PBS and incubated with appropriate fluorescently labeled Alexa-Fluor secondary antibodies (Thermo Fisher; 1:500) for 2 h at room temperature in 0.1% Triton X-100, 2% normal donkey serum, and 1× PBS. Samples were rinsed with 1× PBS and incubated with 1 μg/ml DAPI (Sigma) for 5 min.

### RNA-seq and analysis

Cells were lysed directly in 1 ml of TRIzol (Thermo Fisher) and stored at −80 °C. Once all samples were collected, samples were thawed on ice and RNA was separated with chloroform using Phase Lock Gel tubes (5′). RNA was isolated using the miRNeasy Mini Kit (Qiagen) according to the manufacturer’s protocol. One microgram of each sample was then subject to ribosome depletion, fragmented, and library prepared using the TruSeq Stranded Total RNA Kit with Ribo Zero Gold (Illumina) according to the manufacturer’s protocol and indexed using TruSeq adapters. One hundred base pair paired-end reads were generated for each sample on the Illumina HiSeq 2500 (Case Western Reserve University Sequencing Core, Cleveland, OH, USA). Kruskal–Wallis (non-parametric analysis of variance) was utilized to evaluate genes that change in expression following T3 induction in R. Genes that differ significantly between any two time points were identified (*P* < 0.05) and assigned a “TOM” based on the time point in which the average gene expression across replicates was the highest. In order to obtain a robust signature gene list, we utilized the results from the TOM analysis as well as two additional filters. First, genes with average expression fragments per kilobase of transcript per million (FPKM) <5 at the TOM were excluded. The remaining genes were compared to in vivo sorted OPC and oligodendrocytes^23^. Genes with TOM 72 h post T3 induction were compared to gene expression of newly formed oligodendrocytes, only genes with in vivo expression FPKM >5 were included in the final oligodendrocyte-signature gene set (*n* = 345). Likewise, OPC TOM genes that are expressed FPKM >5 in in vivo OPCs were defined as OPC-signature genes (*n* = 1246).

### Chromosome analysis

Chromosome analysis was performed on 20 G-banded metaphase spreads from each of the four wild-type mESC lines (Cell Line Genetics, Madison, WI, USA). Three of the four lines were karyotypically normal (40,XY). Cell line CBA/Ca demonstrated an abnormal karyotype (40,XY,add(1)(H6)[13]/41,sl, +8[6]). The stemline showed additional material of unknown origin on the distal long-arm of chromosome 1, whereas the sideline showed the abnormality identified in the stemline in addition to a gain of chromosome 8. Despite these abnormalities, no overt differences were observed in this mESC line or derivative OPCs compared to the other three karyotypically normal mESC lines.

### Sequencing *shiverer* deletion

Genomic DNA was isolated from *shiverer* tail tips and Sanger sequenced with the following forward and reverse primers, CAGGGGATGGGGAGTCAGAAGTGAG and ATGTATGTGTGTGTGTGCTTATCTAGTGTA. Sequence transcript was compared to the mouse reference genome in order to identify the *shiverer* deletion sequence.

## Electronic supplementary material

Supplementary Information

Description of Additional Supplementary Files

Supplementary Data 1

## Data Availability

The data that support the findings of this study are available from the corresponding author upon request. RNA-seq datasets were deposited to Gene Expression Omnibus database GSE9744.
