# Supplementary material for: Rapid functional genetics of the oligodendrocyte lineage using pluripotent stem cells
Source: Nat Commun. 2018 Sep 13;9:3708. doi: 10.1038/s41467-018-06102-7 (PMC6137209; doi:10.1038/s41467-018-06102-7)
Supplement: Supplementary file 1 — Supplementary Information [file 41467_2018_6102_MOESM1_ESM.pdf]

Supplementary Information

Lager et al. 2018. Rapid functional genetics of the oligodendrocyte lineage using pluripotent stem cells.

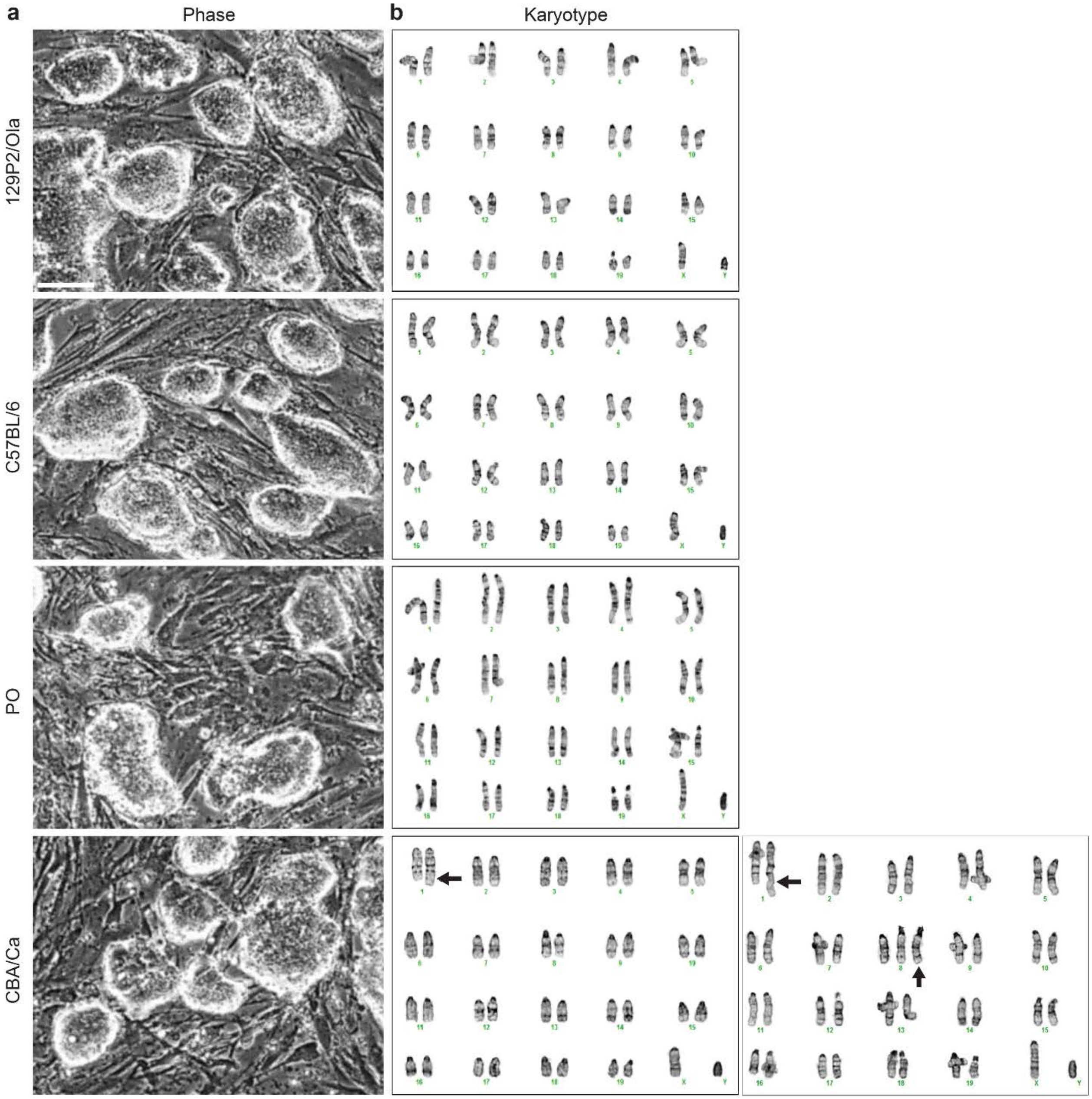

Supplementary Fig. 1 Characterization of mESC lines. **a** Phase contrast images of mESCs from four individual mouse strains—129P2/Ola, C57BL/6, PO, and CBA/Ca. Scale bar, 50 µm. **b** Karyotypes of the same four lines. The mESC line from strain CBA/Ca showed abnormalities on chromosomes 1 and 8 (see Methods).

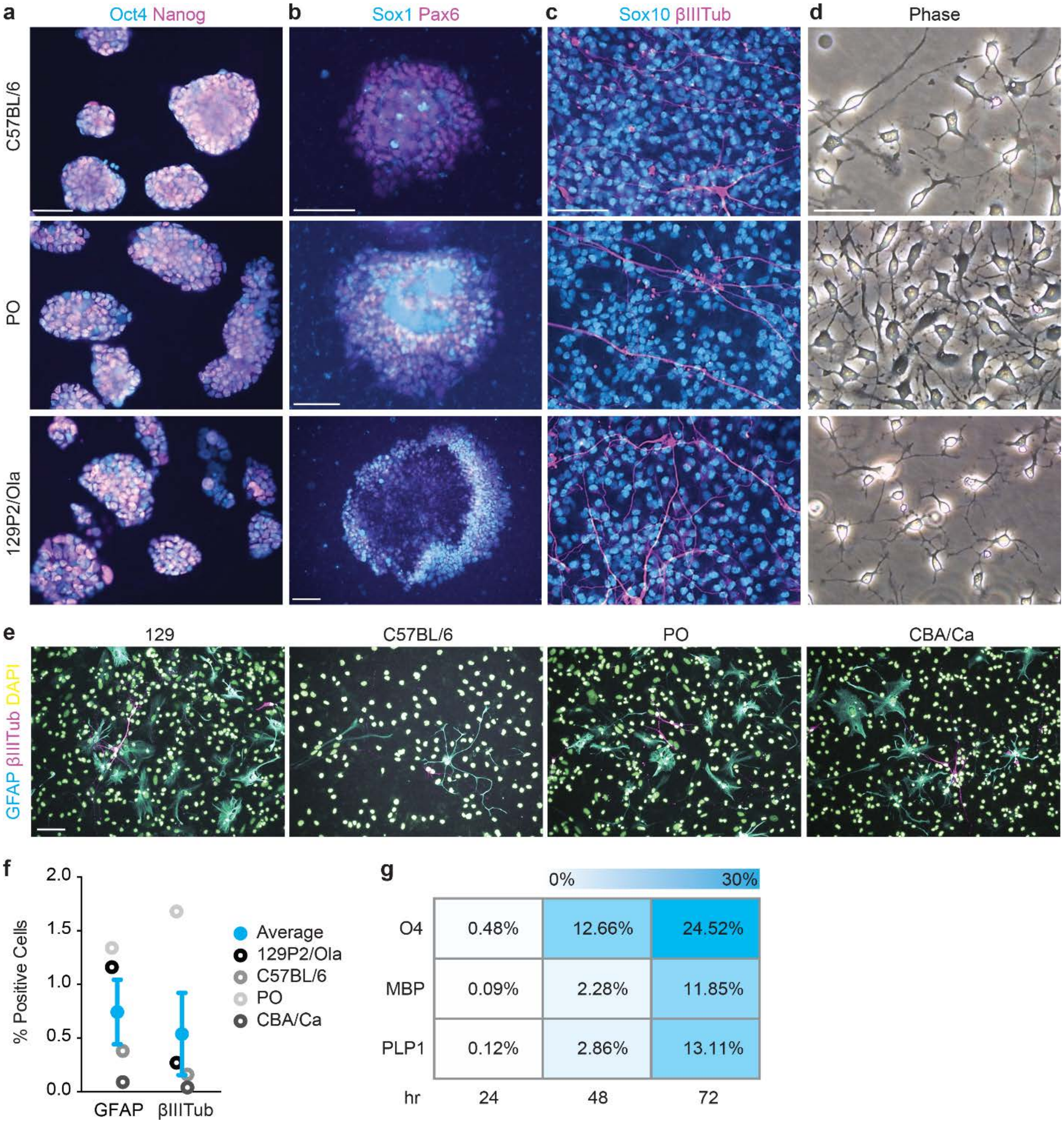

Supplementary Fig. 2 Highly efficient generation of OPCs from multiple mouse strains. **a** Fluorescent images of starting Oct4/Nanog double positive mESCs. Scale bar, 50 µm. **b** Fluorescent images of day 5 differentiated cultures showing Sox1 and Pax6 expression in nascent neuroectoderm. Scale bar, 50 µm. **c** Fluorescent images of passage 1 patterned cultures expressing Sox10, an OPC marker and βIII-Tubulin, a marker of neurons. Scale bar, 50 µm. **d** Phase contrast images of mESC-derived OPCs exhibiting a bipolar morphology. Scale bar, 50 µm. **e** Fluorescent images of passage 1 patterned cultures expressing GFAP, a marker of astrocytes and βIII-Tubulin+, a marker of neurons. Nuclei visualized with DAPI. Scale bar, 50 µm. **f** Quantification of passage 1 cells

expressing GFAP, an astrocyte marker, or βIII-Tubulin+, a neuronal marker. n = 4 independent mESC lines; >76,350 cells scored per well. Data are represented as means ± SEM. **g** Heatmap depicting the percentage of O4, MBP, and PLP1 positive cells at 72 hr post T3 induction. n=4 independent mESC lines; n=7 replicate wells per cell line; >300 cells scored per well. Data are represented as means.

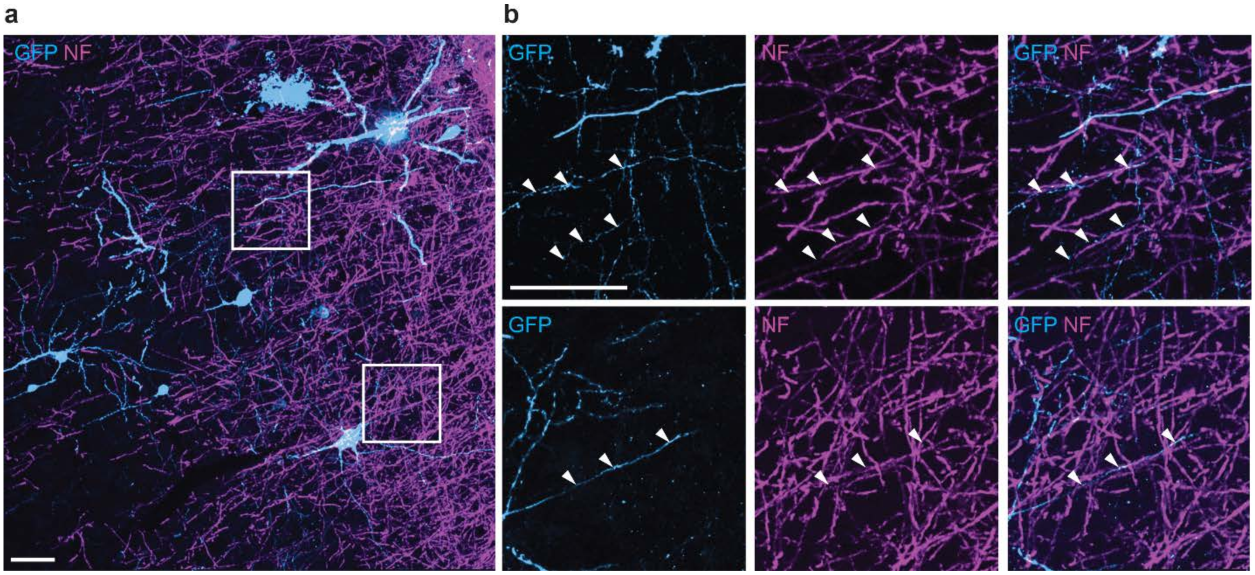

Supplementary Fig. 3 *In vivo* engraftment of *in vitro* derived OPCs. **a** Representative immunofluorescent image of eGFP labeled *in vitro* derived OPCs tracking with NF+ axons 6 weeks after transplantation into the brain of 2 day old athymic nude mice. Scale bar, 50 µm. **b** Insets of immunofluorescent images from Supplementary Fig. 3a demonstrating eGFP+ processes co-localizing with NF+ axons demarcated with white arrow heads. Scale bar, 50 µm.

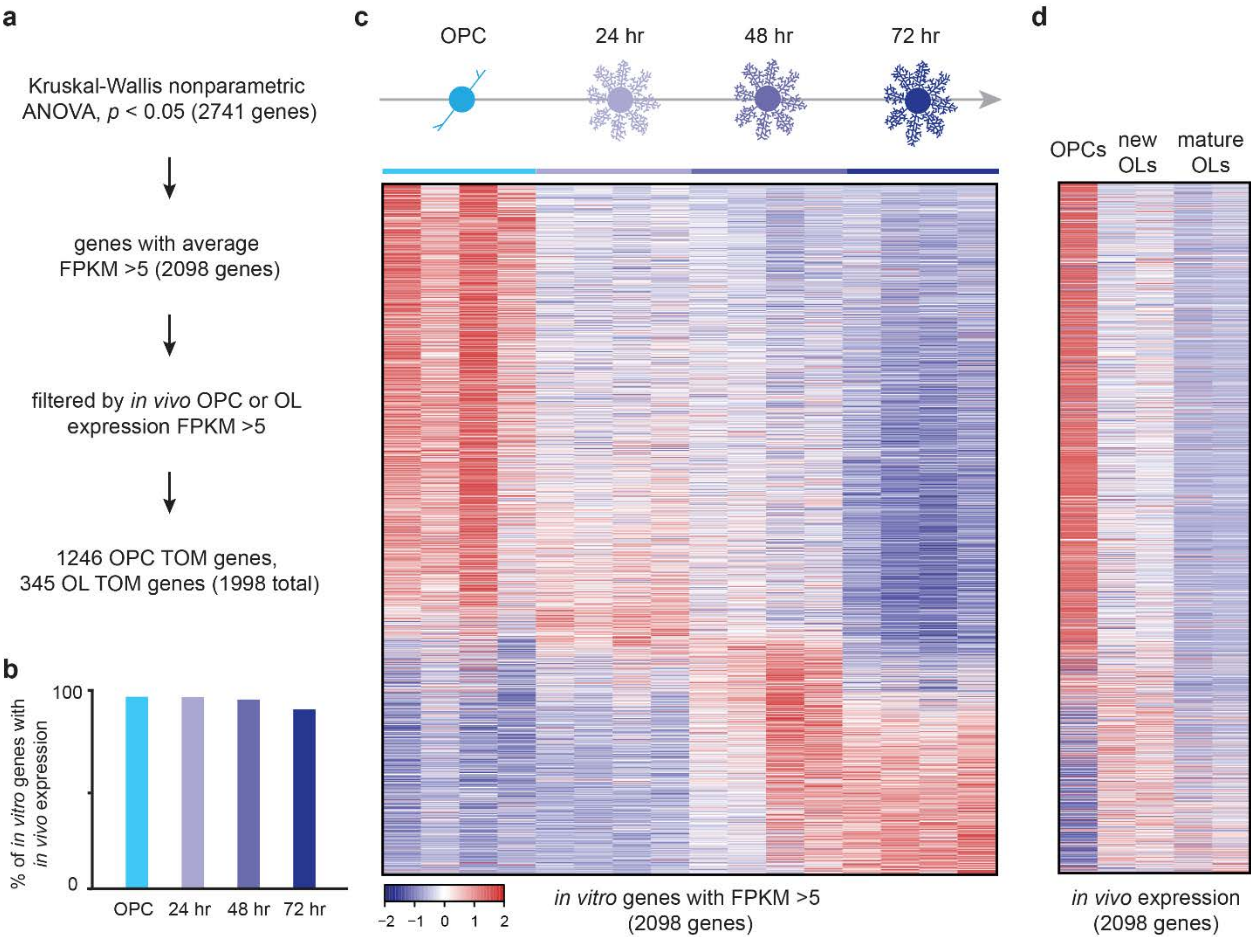

Supplementary Fig. 4 Gene expression profiles of *in vitro* derived OPCs and oligodendrocytes compared to *in vivo* oligodendrocyte lineage cells**. a** Overview flowchart of gene expression analysis. Kruskal-wallis (non-parametic ANOVA) was used to identify genes that differed significantly ($p<0.05$) between any two time points of the OPC differentiation process and assigned time of maximum (TOM). Of those genes, those with FPKM>5 were selected (n=1998 genes) and compared to *in vivo* expression data from OPCs, newly formed oligodendrocytes, or mature oligodendrocytes. Genes that were expressed *in vivo* with an FPKM>5 were subsequently filtered to generate an OPC and OL TOM gene set (n=1246 OPC TOM genes, n=345 OL TOM genes). **b** Quantification of TOM genes at the OPC, 24 hr, 48 hr, and 72 hr post T3 induction stage expressed in *in vivo* oligodendrocytes at FPKM>5. OPC, 96.6%; 24 hr post T3 induction, 96.4%, 48 hr post T3 induction, 95.2%, and 72 hr post T3 induction 90.3%. **c** Row normalized heatmap with genes (rows, n=1998 genes) sorted by time of maximum (TOM) prior to filtering for *in vivo* expression. **d** Heatmap of *in vivo* expressed genes (rows, n=1998 genes) from OPCs, newly formed oligodendrocytes, and mature oligodendrocytes sorted based on row normalization from *in vitro* differentiated OPCs as presented in Supplementary Fig. 4c.
