# Supplementary material for: Rapid functional genetics of the oligodendrocyte lineage using pluripotent stem cells
Source: Nat Commun. 2018 Sep 13;9:3708. doi: 10.1038/s41467-018-06102-7 (PMC6137209; doi:10.1038/s41467-018-06102-7)
Supplement: Supplementary file 2 — Description of Additional Supplementary Files [file 41467_2018_6102_MOESM2_ESM.pdf]

**Description of Additional Supplementary Files**

File Name: Supplementary Data 1
Description: Gene expression profiles of in vitro derived OPCs and oligodendrocytes compared to in vivo oligodendrocyte lineage cells (Excel document).
